# The PAS Domain-Containing Protein HeuR Regulates Heme Uptake in *Campylobacter jejuni*

**DOI:** 10.1128/mBio.01691-16

**Published:** 2016-11-15

**Authors:** Jeremiah G. Johnson, Jennifer A. Gaddy, Victor J. DiRita

**Affiliations:** a Department of Microbiology and Molecular Genetics, Michigan State University, East Lansing, Michigan, USA; b Tennessee Valley Healthcare Systems, Department of Veterans Affairs, Nashville, Tennessee; c Vanderbilt University Medical Center, Department of Medicine, Nashville, Tennessee

## Abstract

*Campylobacter jejuni* is a leading cause of bacterially derived gastroenteritis. A previous mutant screen demonstrated that the heme uptake system (Chu) is required for full colonization of the chicken gastrointestinal tract. Subsequent work identified a PAS domain-containing regulator, termed HeuR, as being required for chicken colonization. Here we confirm that both the heme uptake system and HeuR are required for full chicken gastrointestinal tract colonization, with the *heuR* mutant being particularly affected during competition with wild-type *C. jejuni*. Transcriptomic analysis identified the *chu* genes—and those encoding other iron uptake systems—as regulatory targets of HeuR. Purified HeuR bound the *chuZA* promoter region in electrophoretic mobility shift assays. Consistent with a role for HeuR in *chu* expression, *heuR* mutants were unable to efficiently use heme as a source of iron under iron-limiting conditions, and mutants exhibited decreased levels of cell-associated iron by mass spectrometry. Finally, we demonstrate that an *heuR* mutant of *C. jejuni* is resistant to hydrogen peroxide and that this resistance correlates to elevated levels of catalase activity. These results indicate that HeuR directly and positively regulates iron acquisition from heme and negatively impacts catalase activity by an as yet unidentified mechanism in *C. jejuni*.

## IMPORTANCE

Annually, *Campylobacter jejuni* causes millions of gastrointestinal infections in the United States, due primarily to its ability to reside within the gastrointestinal tracts of poultry, where it can be released during processing and contaminate meat. In the developing world, humans are often infected by consuming contaminated water or by direct contact with livestock. Following consumption of contaminated food or water, humans develop disease that is characterized by mild to severe diarrhea. There is a need to understand both colonization of chickens, to make food safer, and colonization of humans, to better understand disease. Here we demonstrate that to efficiently colonize a host, *C. jejuni* requires iron from heme, which is regulated by the protein HeuR. Understanding how HeuR functions, we can develop ways to inhibit its function and reduce iron acquisition during colonization, potentially reducing *C. jejuni* in the avian host, which would make food safer, or limiting human colonization.

## INTRODUCTION

*Campylobacter jejuni* is a leading cause of gastrointestinal infection worldwide, with a projected 1.3 million annual cases in the United States (1). The prevalence of *C. jejuni* infection is due to its ability to reside asymptomatically within the gastrointestinal tract of avians, especially chickens. During processing of poultry, *C. jejuni* is released from the gastrointestinal tract, contaminating meat products as a result. Patients with *C. jejuni* infection typically develop mild to severe, bloody diarrhea that may be accompanied by fever. Additionally, several postinfectious disorders have been associated with *C. jejuni* infection, including Guillain-Barre Syndrome, postinfectious irritable bowel syndrome, and reactive arthritis (2, 3). Further highlighting these concerns is increasing resistance of *C. jejuni* to the clinically relevant antibiotics azithromycin and ciprofloxacin, prompting the Centers for Disease Control and Prevention to list antibiotic-resistant *C. jejuni* as a “serious threat” to public health (1).

Due to the importance of chickens in human *C. jejuni* infection, much emphasis has been placed on identifying and characterizing factors that are required for colonization of the chicken gastrointestinal tract. Previously, our group has used both signature-tagged mutagenesis (STM) and insertion sequencing (transposon sequencing [Tn-Seq]) approaches to identify colonization determinants of *C. jejuni* (4, 5). The earlier STM approach identified genes involved in chemotaxis and flagellar biogenesis, but several other colonization factors were identified using the Tn-Seq approach. One determinant identified to be reduced by 250-fold in cecal outputs by Tn-Seq was a component of the heme utilization gene cluster *chuB* (Cjj81176_1602), indicating that acquiring iron from heme may be important during colonization of the chicken gastrointestinal tract (4).

Due to the prominent redox potential of Fe^2+^/Fe^3+^, most organisms require iron as a redox cofactor for enzyme complexes. Since the host often restricts iron availability to limit microbial growth—a process termed nutritional immunity—many bacterial species have developed methods for obtaining iron from host molecules, including heme (6). The heme utilization gene cluster of *C. jejuni* consists of genes encoding a predicted TonB-dependent heme receptor (*chuA*), a hemin ABC transporter permease (*chuB*), a hemin ABC transporter ATP-binding protein (*chuC*), and a periplasmic hemin-binding protein (*chuD*) (7). Divergently transcribed from the transport genes is a previously characterized heme oxygenase, *chuZ*, which directly binds heme and degrades it in the presence of an electron donor (7, 8). All components of the heme utilization gene cluster are required for efficient use of heme as a sole iron source, and the ferric uptake repressor (Fur) protein directly binds to the promoter region of the gene cluster, implicating Fur as a *chu* locus regulator (7).

These data are consistent with observations that, in most organisms, intracellular iron levels are tightly regulated to avoid forming toxic hydroxyl radicals and superoxide anions via Haber-Weiss-Fenton catalysis (9). Iron homeostasis specifically impacts the *C. jejuni* response to oxidative stress in two ways: (i) excess iron is associated with repressed catalase production, thereby increasing hydrogen peroxide levels within the cell, and (ii) iron is a required cofactor for periplasmic reductases such as Mfr which interact with H_2_O_2_ to generate reactive oxygen species (ROS) (10, 11). However, *C. jejuni*, like most bacteria, requires nutrient iron to grow and proliferate, leading it to have evolved elaborate regulatory circuits to govern the expression of iron acquisition, metabolism, and efflux. While the above studies indicated that increased iron levels repress *chu* gene expression, likely via Fur binding, no positive regulators of heme and/or iron uptake have been identified. Due to the importance of iron in combating oxidative stress in *C. jejuni*, it would be surprising if *Campylobacter* did not have some mechanism for increasing iron uptake under unfavorable redox states. As such, we hypothesized that *C. jejuni* likely maintains intracellular iron levels using both negative regulation, as seen in previous work with Fur, and positive regulation.

In subsequent Tn-Seq experiments that aimed at finding additional chicken colonization determinants, a candidate regulator was identified, Cjj81176_1389 (unpublished data). This regulator is predicted to contain an N-terminal Per-Arnt-Sim (PAS) domain and a C-terminal helix-turn-helix DNA binding domain. The PAS domain is a ligand-binding domain that interacts with a chemically diverse array of small molecules (12). These domains, while highly divergent in primary sequence identity, maintain a conserved three-dimensional architecture (12). When ligand binds to a PAS domain, the protein either directly initiates a signaling response, or the protein-ligand complex becomes capable of responding to a secondary cue, including gas molecules, photons, or redox potential (12). We reasoned that since the heme utilization gene cluster and Cjj81176_1389 appear to be required for full colonization of the chicken gastrointestinal, and since Cjj81176_1389 may be sensing intracellular redox potential, Cjj81176_1389 could be directly regulating *chu* gene expression.

Here we demonstrate that Cjj81176_1389 mutants have significantly reduced ability for chicken gastrointestinal tract colonization, particularly during competition with wild-type *C. jejuni*. Subsequent transcriptomic analysis found that this regulator, here referred to as HeuR (for **he**me **u**ptake **r**egulator), positively influences *chu* gene expression, as well as other iron uptake systems. Purified HeuR specifically binds the *chuZA* promoter region, indicating it may regulate *chu* gene expression directly. Furthermore, *heuR* mutants are unable to efficiently use heme as the sole iron source and exhibit decreased levels of cell-associated iron. Finally, we show that loss of *heuR* leads to hydrogen peroxide resistance, in turn due to elevated levels of catalase activity.

## RESULTS

### *C. jejuni* strains lacking *heuR* are unable to efficiently colonize the chicken gastrointestinal tract.

**FIG 1:**
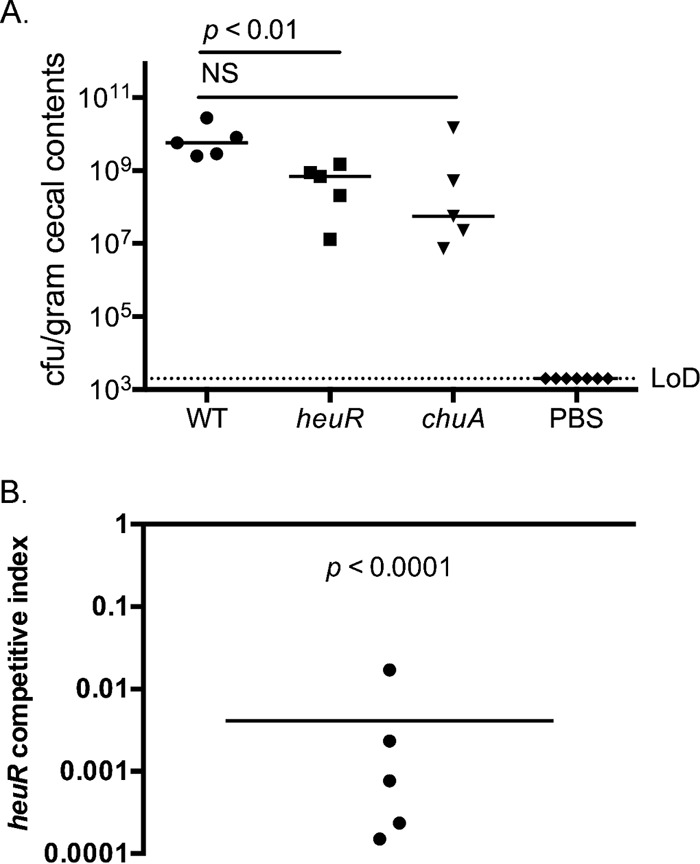
Colonization of day-of-hatch chicks with *heuR* mutants. (A) Monocolonization of day-of-hatch white Leghorn chicks with either wild-type *C. jejuni*, the *heuR* mutant, or the *chuA* mutant. Cecal loads were determined using selective media following a 7-day colonization. The median values are noted and results compared using the Mann-Whitney *U* test. LoD, limit of detection. (B) Competition analysis of wild-type *C. jejuni* versus the *heuR* mutant. Following correction for inocula, the ratio of *heuR* mutant to wild type was determined and is presented as a competitive index. Statistical analysis was performed using a one-sample *t* test against a hypothetical value of 1.

Colonization studies using defined insertion deletion mutants were performed using day-of-hatch chicks. Animals were inoculated with 2.5 × 10^8^ CFU of wild-type *C. jejuni*, 1.7 × 10^8^ CFU of the *heuR* mutant, or 1.9 × 10^8^ CFU of the *chuA* mutant. Following 7 days of colonization, viable bacteria present within cecal samples were enumerated, and the medians were found to be 5.8 × 10^9^ CFU/g for wild-type *C. jejuni*, 6.6 × 10^8^ CFU/g for the *heuR* mutant, and 5.6 × 10^7^ CFU/g for the *chuA* mutant (Fig. 1A). The chicks that were mock infected with phosphate-buffered saline (PBS) did not yield detectable *C. jejuni*. Statistical analysis of the medians revealed that the *heuR* mutant was significantly decreased for colonization, but the *chuA* mutant was not (*P* = 0.1).

Competition analysis using the *heuR* mutant indicated a severe competitive defect of the mutant versus wild-type *C. jejuni*. The *heuR* mutant yielded a mean competitive index value of 0.004 ± 0.007, representing a 250-fold decrease in colonization potential compared to wild-type *C. jejuni* (Fig. 1B). Statistical analysis of this competitive disadvantage was found to be significant.

### Genes involved in inorganic ion transport are underexpressed in the *heuR* mutant.

**FIG 2:**
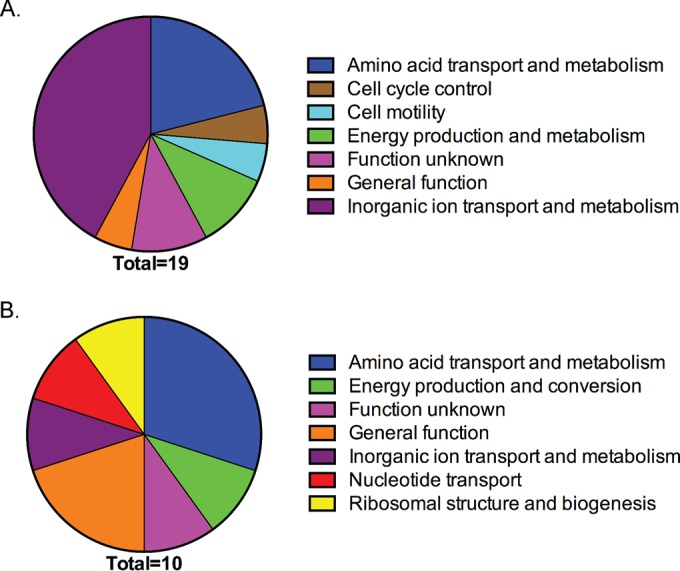
COG analysis of genes differentially expressed in the *heuR* mutant. (A) Identified COGs for genes in Table 1 that were found to be underexpressed in the *heuR* mutant. (B) COGs identified for genes in Table 2 that were overexpressed in the *heuR* mutant.

To determine the extent of the HeuR regulon, we employed transcriptomic analysis of wild-type *C. jejuni* and *heuR* mutant cultures from solid media containing sheep’s blood. Since the conditions required for HeuR-mediated regulation are unknown, we chose these inherently more heterogeneous cultures given that the resolution of transcriptome sequencing (RNA-Seq) would still allow us to observe statistically significant changes. RNA-Seq analysis of the *heuR* mutant identified 43 genes whose transcripts were decreased in abundance by at least 2-fold compared to wild-type *C. jejuni* (Table 1). These values represented statistically significant decreases for all genes identified. The Microbial Genomic context Viewer (MGcV) identified the NCBI GI numbers for 31/43 loci and, using a cluster of orthologous group (COG) analysis, assigned a functional class to 19/31 loci. Of the 19 loci for which a function could be assigned, 8 (42%) were involved in inorganic ion transport and metabolism, including all five *chu* genes (Fig. 2A). Also, amino acid transport and metabolism were well represented with four loci (21%). The MGcV analysis did not identify NCBI GI numbers for the enterochelin system, which was also identified—iron acquisition by this system has been described in *Campylobacter* and multiple organisms (13, 14).

**TABLE 1:** Genes with reduced transcript abundance in the *heuR* mutant

Locus	Predicted function	Fold change (log_2_)	*P* value^a^
CJJ81176_1389	Conserved hypothetical protein	−5.62	4.5E−108
CJJ81176_1601	TonB-dependent heme receptor	−3.34	1.2E−25
CJJ81176_1603	Hemin ABC transporter, ATP binding	−3.32	1.0E−36
CJJ81176_1604	Hemin ABC transporter, periplasmic binding	−3.22	9.3E−33
CJJ81176_1602	Hemin ABC transporter, permease protein	−3.12	2.2E−38
CJJ81176_1704	16S rRNA	−2.48	5.1E−03
CJJ81176_1727	23S rRNA	−2.47	1.8E−04
CJJ81176_1707	23S rRNA	−2.46	2.1E−04
CJJ81176_1714	23S rRNA	−2.45	1.9E−04
CJJ81176_1711	16S rRNA	−2.45	6.0E−03
CJJ81176_1724	16S rRNA	−2.44	5.8E−03
EBG00001201819	Unknown	−2.42	2.3E−04
EBG00001201824	Unknown	−2.42	2.7E−04
EBG00001201849	Unknown	−2.41	6.4E−03
EBG00001201811	Unknown	−2.41	2.6E−04
EBG00001201820	Unknown	−2.40	6.3E−03
EBG00001201839	Unknown	−2.39	8.0E−03
CJJ81176_1386	Conserved hypothetical protein	−2.39	3.6E−13
CJJ81176_1385	Hypothetical protein	−2.33	6.4E−18
CJJ81176_1600	Conserved hypothetical protein	−1.81	8.5E−12
CJJ81176_1623	Putative periplasmic protein	−1.42	4.6E−08
CJJ81176_0471	TonB-dependent receptor, putative	−1.28	2.4E−06
CJJ81176_1333	Flagellin modification protein, PseA	−1.28	2.0E−10
CJJ81176_0761	Conserved hypothetical protein	−1.24	1.8E−07
CJJ81176_0075	Cytochrome *c* family protein	−1.22	5.0E−05
CJJ81176_0235	Citrate transporter, authentic frameshift	−1.20	1.9E−09
CJJ81176_0241	Hypothetical protein	−1.19	3.8E−08
CJJ81176_1443	Hypothetical protein	−1.18	9.4E−06
CJJ81176_0710	Flagellar L-ring protein FlgH	−1.17	8.4E−08
CJJ81176_0760	Hemagglutination domain protein	−1.16	2.1E−04
CJJ81176_0052	Sodium/dicarboxylate symporter	−1.15	1.9E−07
CJJ81176_1353	Enterochelin ABC transporter, ATP binding	−1.15	6.0E−06
CJJ81176_1355	Conserved hypothetical protein	−1.12	6.1E−03
CJJ81176_1354	Enterochelin ABC transporter, periplasmic	−1.11	7.4E−08
CJJ81176_0875	Hypothetical protein	−1.11	7.2E−07
CJJ81176_1622	Hypothetical protein	−1.10	6.6E−07
CJJ81176_0928	Amino acid ABC transporter, periplasmic	−1.10	5.0E−05
CJJ81176_0772	Conserved hypothetical protein	−1.10	4.6E−11
CJJ81176_0067	γ-Glutamyltransferase	−1.04	1.2E−03
CJJ81176_0722	Glutamine synthetase, type I	−1.04	1.5E−05
CJJ81176_1062	Conserved hypothetical protein	−1.04	8.5E−12
CJJ81176_0440	Conserved hypothetical protein	−1.02	7.5E−06
CJJ81176_1332	Imidazole glycerol phosphate synthase	−1.01	2.1E−11

^a^ False discovery rate corrected using the Benjamini-Hochberg method.

In addition to those genes that were found to exhibit reduced transcription in the *heuR* mutant, transcriptomic analysis identified 16 genes with elevated transcript abundance in the mutant (Table 2). The majority of this set included genes belonging to two gene clusters: Cjj81176_1390 to -1394 and Cjj81176_1214 to -1217. MGcV identified NCBI GI numbers for 11/16 genes and assigned functions to 10/11 loci. This group is fairly diverse, with the largest representation belonging to the amino acid transport and metabolism COG (30%) (Fig. 2B).

**TABLE 2:** Genes with elevated transcript abundance in the *heuR* mutant

Locus	Predicted function	Fold change (log_2_)	*P* value^a^
CJJ81176_1390	Endoribonuclease L-PSP, putative	3.49	9.5E−51
CJJ81176_1391	Cryptic C4-dicarboxylate transporter DcuD	3.32	3.1E−43
CJJ81176_1394	MmgE/PrpD family protein	3.19	4.3E−42
CJJ81176_1393	Adenylosuccinate lyase	3.17	5.8E−41
CJJ81176_1392	Cystathionine β-lyase	3.16	5.0E−39
CJJ81176_pVir0034	Hypothetical protein	1.79	2.0E−07
CJJ81176_1217	5,10-Methylenetetrahydrofolate reductase	1.71	3.0E−28
CJJ81176_1216	Methionine synthase MetE	1.71	1.5E−14
CJJ81176_1215	Lipoprotein, NLPA family	1.71	1.4E−12
CJJ81176_1214	Oxidoreductase, 2OG-Fe(II) oxygenase	1.52	7.5E−13
CJJ81176_0271	MCP-domain signal transduction protein	1.40	5.8E−03
EBG00001201809	Unknown	1.08	1.6E−03
EBG00001201810	Unknown	1.05	1.7E−03
EBG00001201838	Unknown	1.05	3.2E−05
EBG00001201844	Unknown	1.03	1.4E−02
CJJ81176_0257	Conserved hypothetical protein	1.01	7.5E−07

^a^ False discovery rate corrected using the Benjamini-Hochberg method.

### Purified HeuR binds to the *chuZA* promoter region.

**FIG 3:**
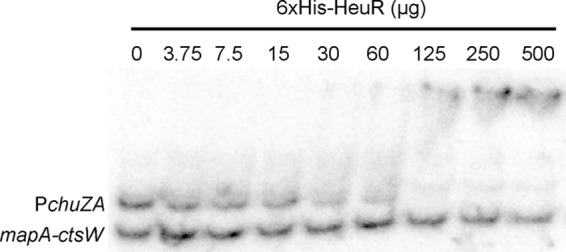
Binding of purified 6×His-HeuR to the *chuZA* promoter region (P*chuZA*). Increasing amounts of purified 6×His-HeuR were added to binding reaction mixtures containing approximately 0.25 nM *chuZA* promoter fragment and the *mapA-ctsW* intergenic region as a nonspecific control.

The putative structure of HeuR includes a C-terminal helix-turn-helix DNA binding domain. Given the transcriptome data demonstrating that *chu* RNA abundance is dependent on HeuR, we hypothesized that HeuR directly regulates *chu* gene expression. Purification of 6×His-tagged HeuR was successful as soluble protein was obtained at concentrations of 4.3 mg/ml. Incubation of purified 6×His-HeuR with radiolabeled *chuZA* promoter and subsequent resolution by native polyacrylamide gel electrophoresis indicated binding of the protein to the *chuZA* probe using 30 µg of 6×His-HeuR (Fig. 3). The shifted species increased in intensity until 125 µg of HeuR was used, and the fragment representing unbound *chuZA* promoter was not detected. These results were in contrast to those obtained for a radiolabeled fragment that represented the unrelated intergenic region between *C. jejuni* 81-176 *mapA* and *ctsW*, which are not regulated by HeuR (based on our RNA-Seq results). Mobility of this probe was relatively unaffected until 500 µg of HeuR was used, when unbound probe intensity was modestly decreased. These results indicate that HeuR binds to a sequence corresponding to the *chuZA* promoter, but not indiscriminately to one other unrelated sequence.

### Mutants of *heuR* are unable to use heme as a source of iron.

**FIG 4:**
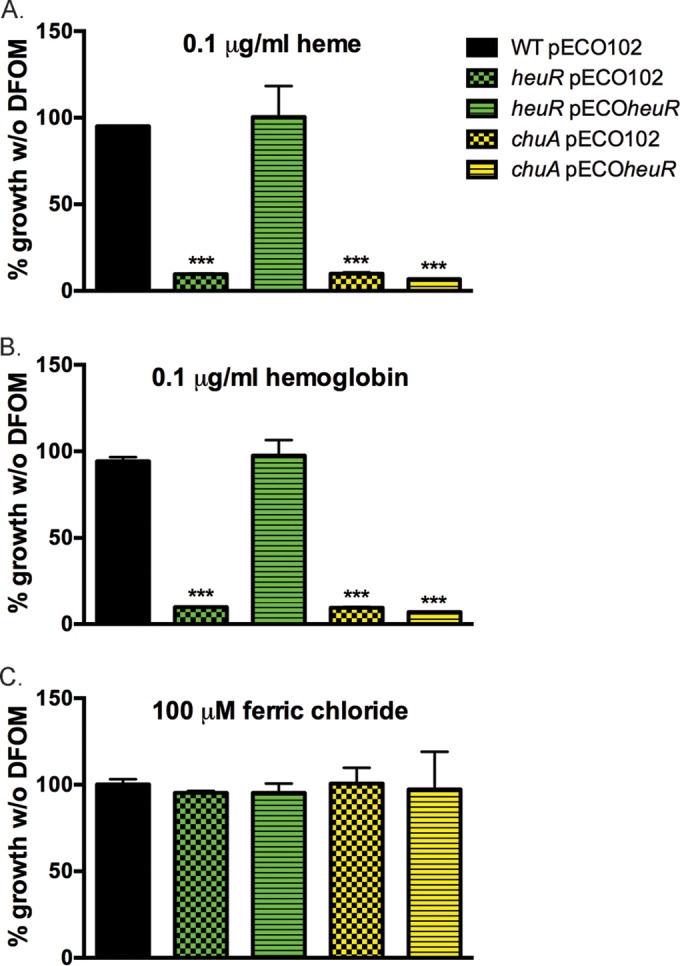
The *heuR* mutant is deficient in using heme as an iron source. (A) Growth of strains in media containing 160 µM desferrioxamine and 0.1 µg/ml purified heme. Values are represented as percentage of growth of the same strains in media without desferrioxamine. (B) Growth of strains in media containing 160 µM desferrioxamine, but with 0.1 µg/ml purified hemoglobin. Values are represented as the percentage of growth of strains in media without desferrioxamine. (C) Control growth of strains in media containing 160 µM desferrioxamine, but with 100 µM ferric chloride. Also represented as the percentage of growth of strains in media without desferrioxamine. Statistical analysis was performed using Student’s *t* test. ***, *P* < 0.0001.

To determine whether decreased expression of the Chu system affected the ability of the *heuR* mutant to use heme as an iron source, cultures of each strain were grown under iron-chelated conditions and supplemented with heme, hemoglobin, or ferric chloride. Both the *heuR* and *chuA* mutants were hypersensitive to iron-limiting conditions compared to wild-type *C. jejuni* (see Fig. S1 in the supplemental material). At 160 µM desferrioxamine (DFOM), the *heuR* mutant with empty vector and the *chuA* mutant with empty vector exhibited greatly reduced growth compared to their behavior in iron-replete medium (Fig. 4A). Heme added to these cultures was unable to rescue growth of the *heuR* and *chuA* mutants. Complementation of the *heuR* mutant with cloned *heuR* restored the ability to use heme as an iron source. In contrast, cloned *heuR* introduced into the *chuA* mutant did not restore heme-dependent growth. Similar results were also seen using whole hemoglobin (Fig. 4B). That iron restriction is the explanation for these phenotypes was supported by the restoration of growth in all strains through the addition of ferric chloride (Fig. 4C).

### Mutation of *heuR* results in decreased cell-associated iron.

**FIG 5:**
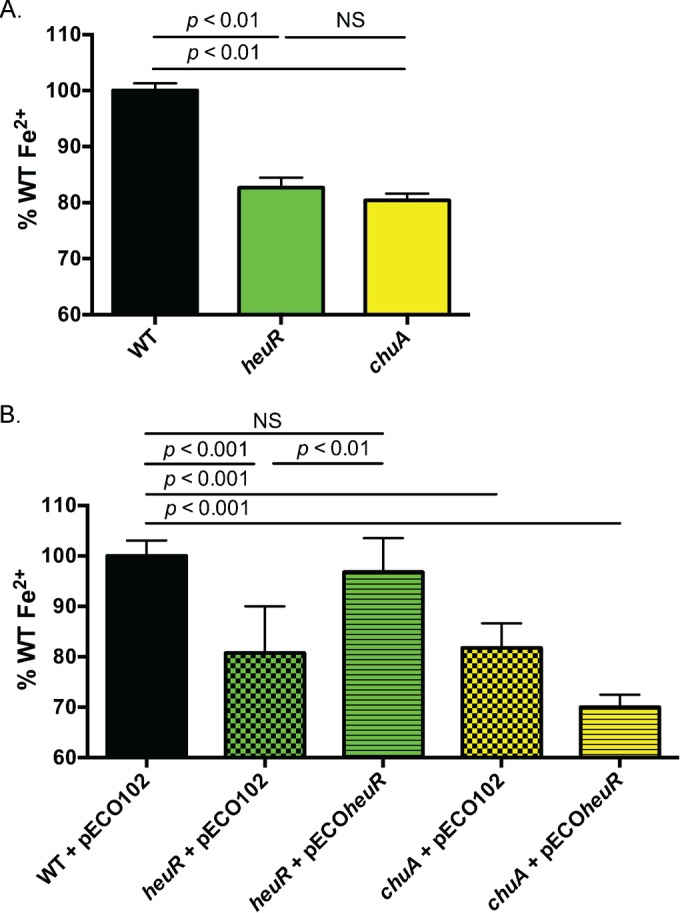
ICP-MS analysis of *C. jejuni* cell-associated iron. (A) Levels of cell-associated iron in wild-type *C. jejuni*, the *heuR* mutant, and the *chuA* mutant. Parts-per-billion iron levels for each strain were expressed as a percentage of the average iron levels observed in wild-type *C. jejuni*. (B) Complementation analysis of iron levels in strains carrying the empty vector control (pECO102) or the complementation construct (pECO*heuR*). Parts-per-billion iron levels for each strain are expressed as a percentage of the average iron levels observed in wild-type *C. jejuni* with empty vector. Statistical analyses were performed using one-way analysis of variance (ANOVA) with Bonferroni’s correction for multiplicity. NS, not significant.

The *heuR* mutant expressed a lower abundance of mRNA from several iron uptake systems (Table 1), suggesting it may play a general role in regulating iron acquisition. To test this, we examined cell-associated iron levels using inductively coupled plasma mass spectrometry (ICP-MS) of wild-type *C. jejuni*, *heuR* mutant, and *chuA* mutant lysates. This analysis demonstrated that iron levels of the *heuR* and *chuA* mutants significantly decreased compared to those of wild-type *C. jejuni* (Fig. 5A).

Iron levels in samples of the *heuR* mutant complemented with cloned *heuR* were similar to those of wild-type *C. jejuni* (Fig. 5B) (*P* = 0.28). Similar to the results of the mutation alone, iron levels in the *chuA* mutant with pECO102 were significantly decreased compared to those of wild-type *C. jejuni*. Consistent with our hypothesis that HeuR regulates iron acquisition through *chu* expression, *w*hen *heuR* was expressed in the *chuA* mutant, cell-associated iron remained significantly decreased compared to that in wild-type *C. jejuni* (Fig. 5B).

### The *heuR* mutant exhibited increased resistance to hydrogen peroxide.

**FIG 6:**
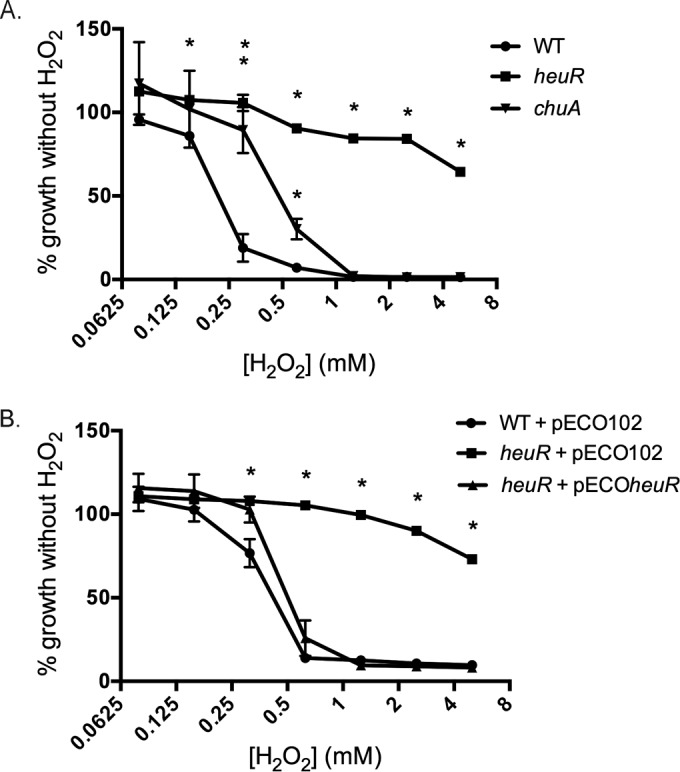
Dose-response analysis of *C. jejuni* mutants grown in the presence of hydrogen peroxide. (A) Growth of wild-type *C. jejuni*, the *heuR* mutant, and the *chuA* mutant in response to increasing concentrations of hydrogen peroxide. (B) Complementation analysis of hydrogen peroxide resistance conferred by the *heuR* mutation. Cells of the wild-type strain with empty vector, the *heuR* mutant with empty vector, or the *heuR* mutant with cloned *heuR* were grown in the presence of increasing hydrogen peroxide concentrations. All growth is expressed as a percentage of that observed for each strain in media alone. Statistical analysis was performed using a Student’s *t* test. *, *P* < 0.01.

We observed a lack of alpha-hemolysis on blood plates on which the *heuR* mutant was grown (data not shown). As alpha-hemolysis is due to the formation of methemoglobin in the presence of hydrogen peroxide, we hypothesized that the *heuR* mutant may degrade hydrogen peroxide more readily than wild-type *C. jejuni*. Support for this came by examining whether the *heuR* mutant could detoxify hydrogen peroxide—which is often done through catalase-mediated degradation—and grow in the presence of the molecule. The *heuR* mutant was incubated in the presence of hydrogen peroxide and found to be resistant. Growth of wild-type *C. jejuni* was generally limited by hydrogen peroxide at concentrations above 0.15 mM and exhibited a 50% inhibitory concentration (IC_50_) value of 0.233 mM (Fig. 6A). In contrast, the *heuR* mutant was unaffected by hydrogen peroxide, with significantly increased growth compared to wild-type *C. jejuni* at concentrations above 0.15 mM. As a result, the IC_50_ value was not experimentally determined and by extrapolation was found to be 5.8 mM. The *chuA* mutant exhibited an intermediate phenotype in the presence of hydrogen peroxide, where growth remained relatively unaffected until concentrations reached 0.60 mM, which was significantly more growth than that of wild-type *C. jejuni*. As such, the *chuA* mutant exhibited an IC_50_ of 0.526 mM, a more than 2-fold increase compared to wild-type *C. jejuni* but approximately 10-fold lower than the *heuR* mutant.

Complementation of the *heuR* mutant with cloned *heuR* resulted in a slight hypersensitivity compared to wild-type *C. jejuni*, with an IC_50_ value of 0.203 mM (Fig. 6B).

### Resistance of the *heuR* mutant to hydrogen peroxide is correlated with increased catalase activity.

We hypothesized that the elevated resistance of the *heuR* mutant to hydrogen peroxide may result from increased catalase activity. To determine whether this was the case, suspensions of strains were normalized and added to 30% H_2_O_2_. In this standard assay, the presence of bacterial catalase leads to conversion of two H_2_O_2_ molecules to two H_2_O molecules and one O_2_ molecule. As such, generation of O_2_ leads to the formation of bubbles, the extent to which correlates to the amount of catalase activity for each strain. The rate of bubble formation can then be qualitatively reported. This assay revealed that wild-type *C. jejuni* carrying empty vector was normally weakly positive (Table 3). In contrast, the *heuR* mutant transformed with empty vector was strongly positive for catalase activity. When *heuR* was reintroduced into the *heuR* background, catalase activity decreased, and the activity of the cells resembled wild-type activity. Also, similar to wild-type *C. jejuni*, the *chuA* mutant remained weakly positive, although the *chuA* mutant did appear to exhibit more catalase activity than wild-type *C. jejuni*.

**TABLE 3:** Catalase activity of *C. jejuni* strains

Strain type (plasmid)	Catalase activity
Wild-type *C. jejuni*	±
*heuR* mutant	++++
*chuA* mutant	+
Wild-type *C. jejuni* (pECO102)	±
*heuR* mutant (pECO102)	++++
*heuR* mutant (pECO*heuR*)	±

## DISCUSSION

Using a Tn-Seq approach, we previously identified several determinants required by *C. jejuni* for efficient colonization of the chicken gastrointestinal tract (4). One determinant was a component of the *Campylobacter* heme utilization gene cluster *chuB*. In this work, we describe a regulator, which we have named HeuR, that is also required for wild-type levels of colonization of the chicken gastrointestinal tract. Additionally, because HeuR is predicted to contain a PAS domain—a protein signature often associated with gas molecule and redox potential sensing—and because it and *chu* are both required for chicken colonization, we hypothesized that HeuR regulates heme uptake through *chu* gene expression.

**FIG 7:**
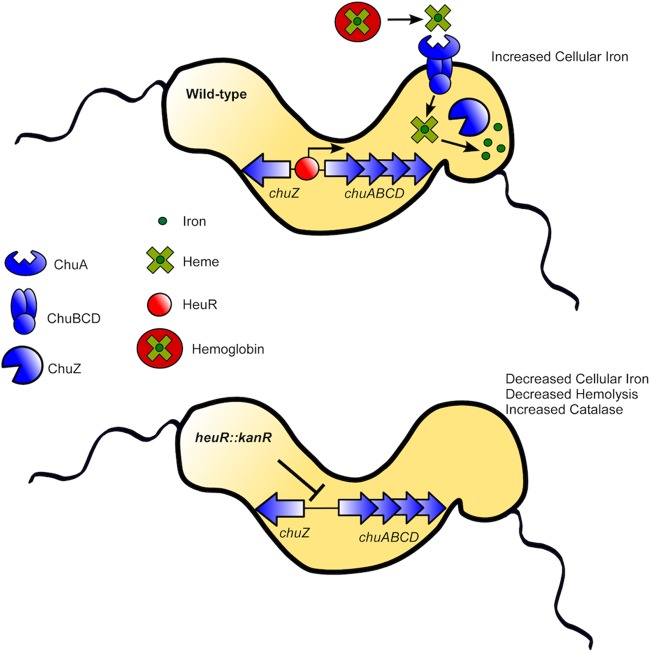
Model of HeuR regulation of the *chuABCDZ* loci and heme homeostasis in *C. jejuni*. Within the vertebrate host, heme is liberated from hemoglobin in an undefined manner. HeuR binds the intergenic region between *chuZ* and *chuABCD* to promote expression of heme acquisition functions. ChuA is the outer membrane heme receptor. The ChuBCD proteins transport heme to the cytoplasm of the cell, where iron is liberated from by the heme oxygenase ChuZ. The *heuR* mutant exhibits decreased expression of the *chuABCDZ* loci, decreased ability to use heme and hemoglobin as a source of nutrient iron, and decreased cell-associated iron levels as a consequence. Additionally, the *heuR* mutant exhibits decreased alpha-hemolysis and increased catalase production.

Recently, HeuR was also found to control expression of Cjj81176_1390, as the gene product was overexpressed in a *heuR* mutant (15). The group that identified this phenotype named HeuR the *Campylobacter* “flagella interaction regulator” (CfiR) due to flagellar interactions that occurred in response to gene disruption. This group further found that the gene product of Cjj81176_1390 forms dimers linked by a disulfide bridge that may be responsive to the redox status of the cell (15). Our observations, recounted below, show that HeuR controls more genes than just those that promote flagellar interactions, including many that may be involved in combating oxidative stress (Fig. 7). As such, we suggest that the designation CfiR does not accurately represent the full regulatory role in the bacterium, and it is our recommendation that the name be changed to HeuR.

By RNA-Seq analysis of a *heuR* mutant strain, we identified several genes whose mRNA products were significantly less abundant, including those in the previously identified *Campylobacter* heme utilization (*chu*) gene cluster. Purified 6×His-HeuR was found to bind specifically to the *chu* promoter, albeit at relatively high levels (125 µg). Previously, regulators containing a PAS domain were found to be inhibited for DNA binding by the PAS domain itself (16). While the reason behind this observation is unknown, it may be due to insufficient copurification of ligand that is required for a higher-efficiency conformation. This may be similar for HeuR and may explain why more protein was required. We are currently working to determine whether this is the case, as well as identifying the specific binding site of HeuR relative to the *chu* promoters.

Additionally, transcript levels of several genes involved in enterochelin transport were decreased in the mutant strain, but were not identified by COG analysis as being involved in ion transport. As a result, this COG analysis is not comprehensive as the number of genes in the HeuR regulon that are involved in iron transport and metabolism is likely underreported. Nevertheless, based on these results, it becomes apparent that HeuR is primarily involved in maintaining sufficient levels of intracellular iron by upregulating systems that can scavenge for iron from diverse sources, which is supported by our ICP-MS data.

In addition to those genes that were underexpressed, transcripts of several were more abundant in the *heuR* mutant. Most of these genes are in two gene clusters: Cjj81176_1390 to -1394 and Cjj81176_1214 to -1217. The former, Cjj81176_1390 to -1394, includes the overexpressed gene (Cjj81176_1390) noted above whose product promotes flagellar interactions, supporting both the previous study and our work (15). The latter gene cluster that is upregulated includes one gene that encodes an additional ion transporter and multiple genes predicted to be involved in methionine biosynthesis. The methionine biosynthetic enzymes encoded by these genes have been shown in other organisms to be sensitive to oxidation/oxidative stress (17). In an oxidative environment, these enzymes may need to be replenished, and their inverse expression with that of iron-scavenging systems may be common. Whether upregulation of these genes is a direct or indirect effect of HeuR is currently unknown.

Growth assays using an iron chelator found that both the *heuR* and *chuA* mutants are reduced for growth under iron-limiting conditions. That this was observed in media without hemoglobin may indicate that the Chu system is partially responsible for the transport of free iron. As such, it would likely be an accessory system since growth was restored when iron was supplemented back, indicating the presence of another iron transport system. When heme and hemoglobin were added to these mutants, growth was not restored, supporting the conclusion that both are required for using heme as an iron source.

Analysis of cell-associated levels of iron showed that the *heuR* mutant is significantly decreased for iron and that these levels resemble those of a *chuA* mutant. Most of the iron reduction observed in these experiments is likely due to decreased heme utilization, as cultures were obtained from media containing sheep’s blood. This is further supported by the observation that complementation with *heuR* restores iron levels in an *heuR* mutant background, but was unable to do so in a *chuA* mutant background. As our data indicate a minor role for HeuR and the Chu system in acquiring iron independently of heme, we are currently determining whether decreased iron is apparent in the *heuR* and *chuA* mutants grown in media without heme or hemoglobin. This will help us determine to what extent other iron sources are impacting cellular iron levels in these mutants.

When the *heuR* mutant was grown on blood plates, differences in the hemolytic pattern relative to wild-type *C. jejuni* became apparent. Typically, *C. jejuni* exhibits alpha-hemolysis on blood plates, likely due to the production of hydrogen peroxide that oxidizes the hemoglobin in the blood to methemoglobin (18). Alpha-hemolysis was not observed in the *heuR* mutant, indicating that this mutant either produces less hydrogen peroxide or exhibits increased catalase activity. The mutant was highly resistant to hydrogen peroxide, which was associated with elevated levels of catalase activity; reintroduction of *heuR* led to both decreased resistance to hydrogen peroxide and catalase activity. Of the genes known to regulate catalase activity in *C. jejuni*—including *ahpC*, *fur*, *katA*, and *perR*—none was found to be significantly up- or downregulated in the *heuR* mutant (data not shown). Also, since iron levels are similar in the *chuA* mutant, it is unlikely that iron levels are responsible for the elevated catalase activity. Thus, we hypothesize that HeuR regulates the production of an unknown factor that perhaps posttranscriptionally regulates the activity of *C. jejuni* catalase. We are currently working to determine what this factor is.

The *heuR* mutant exhibited significant decreases in colonization of the chicken cecum. This leads to the following two questions. (i) Is this system mainly required for combating oxidative stress in the cecum, or is it used for other cellular processes? (ii) Is the system acquiring iron from a heme source, or does it serve the dual purpose of acquiring free iron from the environment?

Oxidative stress in the cecum may come from a variety of sources, like metabolism by the intestinal microbiota or from the generation of reactive oxygen species due to intestinal inflammation. While the *Campylobacter*-chicken relationship is often considered to be commensal in nature, *C. jejuni* colonization of the chicken gastrointestinal tract may result in low levels of transient inflammation (19–21; L. M. Davis and V. J. DiRita, unpublished observation). Also, iron acquisition systems like Chu may be more important for acquiring iron that can be used in other cellular processes, including energy generation and DNA replication (6). Based on the results from the colonization studies, either scenario is possible. During monoinfection, the *heuR* mutant does not need to compete for iron with wild-type *C. jejuni* and should colonize to relatively high levels, which is what we observe. Conversely, during competition, the *heuR* mutant would need to compete with wild-type *C. jejuni* for resources and would be at a disadvantage for colonization—the observed phenotype. We are currently working to determine whether decreasing oxidative stress in the chicken rescues colonization of the *heuR* mutant or whether other processes, like energy production or DNA replication, are affected. Since they are not mutually exclusive, it is possible that both scenarios are supported.

Second, the question arises as to whether *C. jejuni* uses the Chu system to acquire iron from either/or a heme or nonheme source. It is currently unknown how much heme chickens consume in a commercial diet, and we could find no data on heme availability in their natural diet. With the exception of higher plants that can produce heme in the plastids, the absence of heme molecules in most grains, plants, and insects makes it less obvious how chickens would have access to heme-containing molecules (22). The likelihood then becomes that *C. jejuni* uses this system to scavenge heme either from a microbial source or from the host. The *C. jejuni* 81-176 genome encodes all heme biosynthetic enzymes, with the exception of homologs for protoporphyrinogen oxidase (*hemY* or *hemG*). As such, in an iron-rich environment, *C. jejuni* can likely synthesize its own heme. It could then use the heme uptake system to recover heme from other *Campylobacter* cells or from heme-producing residents of the microbiota. Finally, it remains possible that this system is able to transport free iron. While the heme receptor and heme oxygenase (ChuA and ChuZ, respectively) would likely not be needed for free iron transport, the accompanying ABC transport system (ChuBCD) may be promiscuous and function similar to other iron transporters. It should be noted that growth of both the *heuR* and *chuA* mutants could be rescued in chelator-treated media by the addition of iron. This suggests that other iron transport systems are active, at least *in vitro*, so the requirement of the Chu system for *in vivo* iron transport appears unlikely. Regardless, both scenarios are supported by the colonization assays since a mutant deficient for uptake of either heme or nonheme iron would be at a disadvantage in the host. We are currently investigating whether the ChuBCD transporter can translocate nonheme iron into *C. jejuni.*

Campylobacters, like most organisms, have evolved tight regulation on iron acquisition. This is largely due to the observation that iron is a required cofactor for numerous cellular processes but also has the capacity to participate in the formation of reactive oxygen species, leading to oxidative stress within the bacterial cell (23). HeuR participates in controlling the utilization of alternate iron sources. The canonical ferric uptake regulator (Fur) represses iron acquisition under conditions where iron is abundant and readily available and also promotes resistance to both acid and oxidative stress (24, 25). Fur exists as a monomer under low-iron conditions, but when iron is present, Fur dimerizes and binds to discrete locations, termed “Fur boxes” to alter gene expression. PerR is another iron-responsive regulator that contributes to peroxide and superoxide stress defense (26–28). Thus, *C. jejuni* has evolved separate but overlapping regulons controlled by HeuR, Fur, and PerR to aid the cellular response to stimuli, including changes in iron availability and cognate perturbations in cellular oxidative stress (29).

Finally, the question also arises as to the impact of HeuR and the Chu system on dissemination of *C. jejuni* to extraintestinal sites like the spleen and/or liver in poultry (30). Similarly, this system may also be important in human infection as *C. jejuni* has been shown to infect the blood, liver, and pancreas of patients, causing bacteremia, hepatitis, and pancreatitis, respectively (31–33). These sites, compared to the gut, are presumably more restricted for free iron, and the ability of *Campylobacter* to acquire iron from a heme source may be important during these infections.

In conclusion, our work demonstrates that HeuR is a transcriptional regulator that binds to the promoter region of *chuZA* and induces expression of bacterial factors which play a role in inorganic ion transport. As a result of HeuR activity, *C. jejuni* elaborates proteins that aid in the utilization of both heme and hemoglobin as alternate sources of nutrient iron, thereby elevating cell-associated iron in response to iron restriction via nutritional immunity. Lack of HeuR promotes resistance to peroxide, a phenotype that is correlated with elevated catalase production. Together, these results reveal that HeuR is critical for colonization of the gastrointestinal tract of an animal host by promoting metal homeostasis and regulating cellular processes, which are critical for pathogenesis.

## MATERIALS AND METHODS

### Day-of-hatch chick colonization.

Day-of-hatch white Leghorn chickens were inoculated by oral gavage with 100 µl of an approximately 1 × 10^9^-CFU/ml bacterial suspension in sterile phosphate-buffered saline (PBS) (10^8^ CFU). Colonized chickens were housed for 7 days and euthanized, and cecal contents were collected. Cecal contents were homogenized in sterile PBS, and serial dilutions were plated on *Campylobacter* selective media: Mueller-Hinton (MH) agar containing 10% sheep’s blood, cefoperazone (40 µg/ml), cycloheximide (100 µg/ml), trimethoprim (10 µg/ml), and vancomycin (100 µg/ml). Plates were grown for 48 h under microaerobic conditions, and colonies were enumerated. Statistical analysis of the medians was performed using a Mann-Whitney test.

For the competition experiment, equal numbers of wild-type *C. jejuni* cells and the *heuR* mutant were mixed, and approximately 1 × 10^8^ of each were used to inoculate day-of-hatch chicks by oral gavage. Harvest of cecal contents was done as described above, except serial dilutions were plated on *Campylobacter* selective media with and without kanamycin (100 µg/ml). Competitive index values were calculated following correction for inoculum amounts by dividing the number of *heuR* mutants by the number of wild-type bacteria. Statistical analysis was done using a one-sample *t* test against a hypothetical value of 1.

All chicken experiments and protocols were approved by the Institutional Animal Care and Use Committee in the Animal Care Program at Michigan State University.

### RNA-Seq analysis.

Triplicate cultures of wild-type DRH212 and the *heuR* mutant were grown on MH agar supplemented with 10% whole sheep’s blood and 10 µg/ml trimethoprim overnight at 37°C under microaerobic conditions. Cells were harvested and grown on new media for a second night using the same conditions. Following growth, cells were harvested from each plate, and RNA was extracted using a previously described protocol (34). DNA was degraded using Turbo DNA-free (Thermo) according to the manufacturer’s instructions, and removal was confirmed by conventional PCR. RNA quality was assessed using an Agilent 2100 Bioanalyzer, and libraries were generated using the TruSeq RNA library preparation kit (Illumina). Libraries were sequenced using an Illumina HiSeq 2000 formulated for single-end, 50-nucleotide reads. Analysis of sequence data was performed using SPARTA (Simple Program for Automated reference-based bacterial RNAseq Transcriptome Analysis) (35). Briefly, raw reads were trimmed and assessed for quality using Trimmomatic and FastQC, respectively (36). Trimmed reads were mapped to the *C. jejuni* 81-176 reference genome using Bowtie, and expression levels were determined by HTSeq (37, 38). Finally, differential expression analysis was performed using edgeR (39). Cluster of orthologous group (COG) identification of selected genes was performed using the Microbial Genomic context Viewer (MGcV).

### Protein purification.

The gene *heuR* was amplified by PCR from *C. jejuni* 81-176 genomic DNA using primers 5′ Cjj1389 BamHI and 3′ Cjj1389 SalI. The PCR fragment was subcloned into pGEM-T Easy using the manufacturer’s protocol and sequenced using primers T7 and SP6. Fragments that were confirmed to have maintained the full nucleotide sequence of *heuR* were excised using BamHI and SalI and ligated into the expression vector pQE-30 (Qiagen). Ligation products were transformed into *Escherichia coli* NEB C3013, and insertion was confirmed using a restriction digest of purified plasmids. Induction of 6×His-HeuR using 1 mM IPTG (isopropyl-β-d-thiogalactopyranoside) was confirmed for six constructs by SDS-PAGE and Coomassie staining. One strain was used for protein purification where a 1-liter culture was grown to the mid-log phase and induced for 3 h at 37°C using 1 mM IPTG. Cells were pelleted at 8,000 rpm for 20 min at 4°C and resuspended in 20 ml lysis buffer (pH 8.0) (50 mM NaH_2_PO_4_, 300 mM NaCl, 10 mM imidazole). The cell suspension was passed three times through a French press, and cell debris was removed by centrifugation at 8,000 rpm for 20 min at 4°C. The cleared lysate was added to 5 ml of Ni nitrilotriacetic acid (NTA) agarose that was preequilibrated with lysis buffer and rocked overnight at 4°C. This resin-protein mixture was packed by gravity into a chromatography column, and lysate was passed through and collected. Nonspecific proteins were removed by washing the resin bed twice with 5 column volumes (25 ml) of wash buffer (pH 8.0) (50 mM NaH_2_PO_4_, 300 mM NaCl, 20 mM imidazole). Bound protein was eluted four times using 1 ml of elution buffer (pH 8.0) (50 mM NaH_2_PO_4_, 300 mM NaCl, 250 mM imidazole). Successful purification of 6×His-HeuR was verified by SDS-PAGE and Coomassie staining. Fractions containing the largest amount of HeuR were pooled and dialyzed against storage buffer (50 mM Tris [pH 7.5], 150 mM NaCl, 0.5 mM EDTA, 0.02% Triton X-100, 2 mM dithiothreitol [DTT], 50% glycerol). Concentrations of 6×His-HeuR were determined using a bicinchoninic acid (BCA) protein assay (Pierce), and aliquots of protein were stored at −80°C.

### Electrophoretic mobility shift assays.

A specific DNA probe was generated from the *chuZA* promoter region, and a nonspecific probe was generated from the *mapA*-*ctsW* intergenic region by PCR of *C. jejuni* 81-176 genomic DNA. Amplicons were purified using a PCR purification kit (Qiagen) and subsequently end labeled with [γ-^32^P]ATP using T4 polynucleotide kinase (PNK) (New England Biolabs). DNA binding reaction mixtures containing radiolabeled specific (*chuZA* promoter) and nonspecific (*mapA-ctsW* intergenic) probes at approximately 0.25 nM, DNA binding buffer (10 mM Tris, pH 7.5, 50 mM KCl, 1 mM EDTA, 1 mM DTT, 5% glycerol), 25 ng/µl poly(dA-dT), and 100 µg/ml BSA were incubated for 5 min at room temperature. Purified 6×His-HeuR was diluted 2-fold in binding buffer, and 1 µl containing 500, 250, 125, 60, 30, 15, 7.5, or 3.75 µg of protein was added to individual 19-µl binding reaction mixtures (20 µl total). These reaction mixtures—and a control reaction mixture without 6×His-HeuR—were incubated for 15 min at room temperature and subsequently resolved on a 5% polyacrylamide native Tris-glycine gel at 4°C. Gels were imaged using a Typhoon phosphoimager.

### Hemoglobin and heme growth assays.

For bacterial growth assays, *C. jejuni* strains were grown overnight on tryptic soy agar plates supplemented with 5% sheep blood and appropriate antibiotics (chloramphenicol and/or kanamycin). The following day, plate cultures were utilized to inoculate 5 ml of Mueller-Hinton broth supplemented with appropriate antibiotics. These liquid cultures were incubated under shaking conditions at 37°C overnight. The following day, cultures were diluted 1:10 in fresh Mueller-Hinton broth (40) supplemented with appropriate antibiotics (chloramphenicol and/or kanamycin) alone (medium alone) or supplemented with a 40, 80, or 160 µM concentration of the iron chelator desferrioxamine mesylate (Sigma Aldrich) alone or in combination with 0.1 µg/ml of purified human hemoglobin (1) or 0.1 µg/ml of hemin at 37°C in room air supplemented with 5% CO_2_. Bacterial growth was measured using a spectrophotometric reading at 600 nm (OD_600_). Statistical analysis was performed using a Student’s *t* test.

### ICP-MS analysis.

Cultures of wild-type *C. jejuni* DRH212, the *heuR* mutant, and a *chuA* mutant were grown overnight at 37°C under microaerobic conditions on MH agar with 10% whole sheep’s blood containing either 10 µg/ml trimethoprim or 100 µg/ml kanamycin (for mutant strains). For complementation analysis, the wild-type strain with pECO102, the *heuR* mutant with pECO102, the *heuR* mutant with pECO*heuR*, the *chuA* mutant with pECO102, and the *chuA* mutant with pECO*heuR* were grown on MH agar with 10% sheep’s blood containing either chloramphenicol (30 µg/ml) alone (for the wild type) or chloramphenicol and kanamycin (100 µg/ml) (for the plasmid-containing mutants). Cultures were grown overnight under microaerobic conditions at 37°C. Strains were passed for a second night on fresh media and harvested for iron quantification. Approximately 1,010 cells were washed three times with 5 ml and resuspended in 500 µl of molecular-grade, distilled water. Cells were solubilized overnight at 50°C in 1 ml 50% nitric acid, followed by the addition of 9 ml of water.Extracts were analyzed for 57Fe using a Thermo Scientific ICAP Q quadrupole inductively coupled plasma mass spectrometer in kinetic energy dispersion mode. Instrument calibration was done with aliquots of an Fe synthetic standard solution diluted at 2.5, 10, and 100 ppb and natural water standards NIST SRM 1640 and 1643e. A solution of 100 ppb Fe was analyzed every 5 to 6 samples to monitor instrumental drift; all samples were corrected for linear drift. The oxide formation rate was determined to be <2% by measuring CeO/Ce, and the double-charged cation formation rate was found to be <3% by measuring ^137^Ba^2+^/^137^Ba from a solution with approximately 1 ppb Ce and 1 ppb Ba.

Parts-per-billion levels for each strain were normalized to those observed for the wild-type equivalent in each experiment and the standard deviation was plotted. Statistical analysis was performed using a Student’s t test.

### Hydrogen peroxide dose-response assays.

Cultures of wild-type *C. jejuni*, the *heuR* mutant, and the *chuA* mutant were grown overnight at 37°C under microaerobic conditions on MH agar with 10% sheep’s blood. Typically, *C. jejuni* exhibits alpha-hemolysis on blood plates, likely due to the production of hydrogen peroxide that oxidizes the hemoglobin in the blood to methemoglobin. Strains were passed for a second night on new media under the same conditions. Cells were harvested into MH broth and normalized to an optical density at 600 nm (OD_600_) of 0.05. These suspensions were added to equal volumes (100 µl:100 µl) of 2-fold dilutions of MH broth with hydrogen peroxide in a 96-well plate. Hydrogen peroxide concentrations ranged from 10 mM to 0.16 mM. This dilution resulted in bacterial concentrations of 0.025 OD units and hydrogen peroxide concentrations ranging from 5 mM to 0.08 mM. These cultures were grown statically for 48 h at 37°C under microaerobic conditions followed by resuspension of bacteria and recording of terminal OD_600_ values. IC_50_ values were calculated using nonlinear regression analysis of normalized responses with variable slopes in GraphPad Prism 6. Statistical analysis of differences between strains at each concentration of hydrogen peroxide was performed using a Student’s *t* test.For complementation analysis of the *heuR* phenotype, the complemented strains used for ICP-MS analysis were subjected to the same hydrogen peroxide dose-response assay as the strains described above. Statistical analysis was also performed using a Student’s *t* test.

### Catalase assays.

Cultures of wild-type *C. jejuni*, the *heuR* mutant, and the *chuA* mutant were grown overnight at 37°C on MH agar with 10% sheep’s blood under microaerobic conditions. These strains were passed for a second night on new media under the same conditions, and cells were harvested into sterile PBS. Care was given to avoid contaminating the suspensions with media since blood exhibits catalase activity. Suspensions were normalized to an OD_600_ of 1.0, and 10 µl of suspension was added to 10 µl of 30% hydrogen peroxide (J. T. Baker) on a glass slide. Reactions were performed in triplicate and qualitatively noted.

## SUPPLEMENTAL MATERIAL

Figure S1  Dose-response analysis of desferrioxamine. *C. jejuni* strains grown in the presence of increasing amounts of desferrioxamine (DFOM). Growth at each concentration of DFOM is expressed as a percentage of growth of that strain in media alone. Download Figure S1, TIF file, 0.5 MB
