# Supplementary figures and images for: The PAS Domain-Containing Protein HeuR Regulates Heme Uptake in Campylobacter jejuni
Source: mBio. 2016 Nov 15;7(6):e01691-16. doi: 10.1128/mBio.01691-16 (PMC5111405; doi:10.1128/mBio.01691-16)

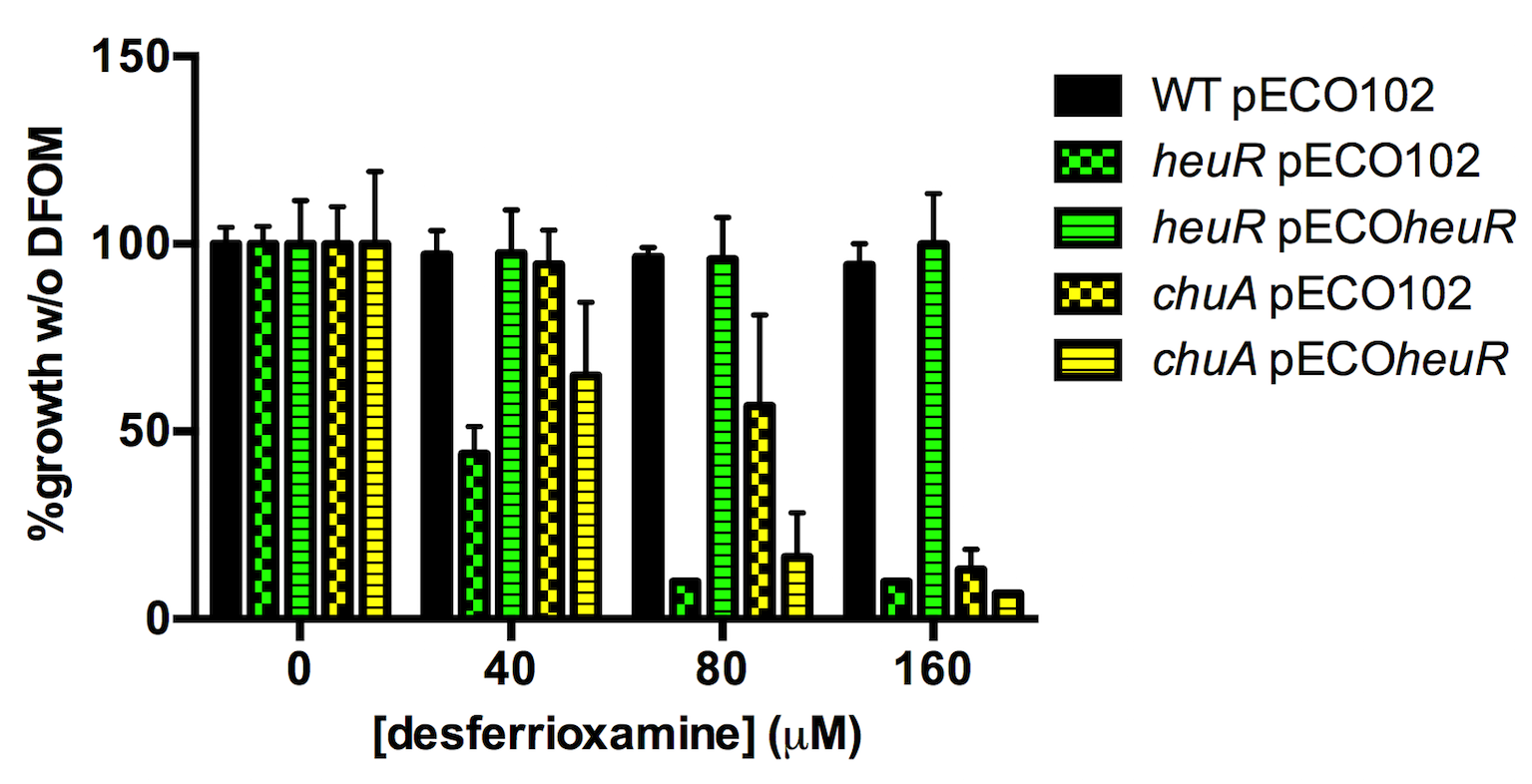

Supplement: Figure S1 — Dose-response analysis of desferrioxamine. C. jejuni strains grown in the presence of increasing amounts of desferrioxamine (DFOM). Growth at each concentration of DFOM is expressed as a percentage of growth of that strain in media alone. Download [file mbo006163071sf1.tif]
